# Disentangling the seasonality effects of malaria transmission in the Brazilian Amazon basin

**DOI:** 10.1098/rsos.231764

**Published:** 2024-07-03

**Authors:** Naiara C. M. Valiati, Benjamin Rice, Daniel A. M. Villela

**Affiliations:** ^1^ National School of Public Health Sergio Arouca, Fundação Oswaldo Cruz, Rio de Janeiro, Brazil; ^2^ Department of Ecology and Evolutionary Biology, Princeton University, Princeton, NJ, USA; ^3^ Program of Scientific Computing, Fundação Oswaldo Cruz, Rio de Janeiro, Brazil; ^4^ Center for Health and Wellbeing, School of Public and International Affairs, Princeton University, Princeton, NJ, USA

**Keywords:** malaria, seasonality, mathematical model

## Abstract

The evidence of seasonal patterns in malaria epidemiology in the Brazilian Amazon basin indicates the need for a thorough investigation of seasonality in this last and heterogeneous region. Additionally, since these patterns are linked to climate variables, malaria models should also incorporate them. This study applies wavelet analysis to incidence data from 2003 to 2020 in the Epidemiological Surveillance System for Malaria (SIVEP-Malaria) database. A mathematical model with climate-dependent parametrization is proposed to study counts of malaria cases over time based on notification data, temperature and rainfall. The wavelet analysis reveals marked seasonality in states Amazonas and Amapá throughout the study period, and from 2003 to 2012 in Pará. However, these patterns are not as marked in other states such as Acre and Pará in more recent years. The wavelet coherency analysis indicates a strong association between incidence and temperature, especially for the municipalities of Macapá and Manaus, and a similar association for rainfall. The mathematical model fits well with the observed temporal trends in both municipalities. Studies on climate-dependent mathematical models provide a good assessment of the baseline epidemiology of malaria. Additionally, the understanding of seasonality effects and the application of models have great potential as tools for studying interventions for malaria control.

## 1. Introduction

In Brazil, there is significant concern regarding malaria, a disease with over 42 million people at risk and 142 124 cases reported to authorities in 2021, as documented by Brazil’s Epidemiological Surveillance Information System (SIVEP) [1], which handles malaria notification data in a specific module named SIVEP-Malaria. These cases primarily consist of 83.7% *Plasmodium vivax* infections, 16.2% *Plasmodium falciparum* infections and 0.1% attributed to *Plasmodium malariae* or remain unidentified [2], and around 76% in the American continent [3].

While successful efforts to reduce malaria cases occurred between 2005 and 2015, recent years have seen a troubling resurgence, particularly in *P. vivax* cases, as noted in a study by Lana *et al*. [4]. This resurgence is a complex issue, as indicated by Ayala *et al*. [5], and is likely driven by factors such as increased agricultural activities, outdoor exposure, human mobility and ongoing deforestation. These concerning trends are mainly observed in the northern region of Brazil, which includes the Amazon basin. This situation underscores the challenges faced by malaria control programmes in this region and their negative impact on socio-economic and environmental conditions.

To address this resurgence, a new national plan has been devised, aiming to eliminate malaria in four phases: (i) reducing the number of cases to fewer than 68 000 by 2025, (ii) eliminating *P. falciparum* malaria cases and malaria-related deaths by 2030, (iii) achieving zero malaria cases and deaths by 2035, and (iv) preventing the reintroduction of malaria from 2035 onwards [6]. Recent reports, however, highlight the unequal distribution of malaria cases across different Brazilian states, with complex and varied trends in recent years [7].

Given the geographic size and heterogeneity of the Amazon region, it is crucial to consider seasonality effects. Additionally, the Amazon region faces the challenge of co-transmission of both *P. vivax* and *P. falciparum* [4,8,9], each with distinct epidemiological dynamics. This adds complexity to the malaria situation in Brazil. Cross-border factors also play a role in this context [10,11], emphasizing the need for a comprehensive understanding of the drivers behind the surge in cases, especially as a malaria vaccine is not yet a public health reality [12]. Fortunately, the SIVEP-Malaria database of Brazil provides information on the identified *Plasmodium* species for each notified case. Malaria is a vector-borne disease, and studying its transmission dynamics requires accounting for factors influencing vector ecology, such as climate variables like temperature and rainfall, which impact various aspects of malaria incidence [13–16]. The goal here is to study the seasonality effects of *P. vivax malaria* observed in the Brazilian Amazon basin, employing climate-dependent modelling. Other aspects such as the transmission cycles of *P. falciparum* and the border effects were not considered.

In this study, we use wavelet analysis to better understand the seasonality effects of malaria in the Brazilian Amazon basin, a technique used in prior research on malaria and diseases in other regions [17–19]. To deepen our understanding of how climate variables influence malaria dynamics, we also conduct coherence analysis through wavelets concerning malaria notification data [20–23]. This approach has been used by numerous researchers to examine the association between climate variables (e.g. temperature and rainfall) and malaria cases and hospital admissions in different countries [17–19,23–28]. The wavelet analysis allows us to assess the non-stationary spatial and temporal dynamics of diseases in relation to climate variables, aiding in the identification of epidemic periodic cycles. Moreover, it enables us to study the relationship between a disease time series and climate data.

Finally, we integrate the findings from wavelet and coherence analyses into a mathematical model, extending a recent model developed by Ayala & Villela [29]. This model accounts for sensitive and resistant strains of malaria and applies climate variables to its parameters to describe transmission dynamics in municipalities within the Amazon region. In summary, our study provides a comprehensive analysis of the multifaceted malaria situation in Brazil, combining epidemiological data, climate variables and mathematical modelling to gain insights into the dynamics of this vector-borne disease in the Amazon basin.

## 2. Methods

### 2.1. Study area and data

The Brazilian Amazon basin is an extensive geographical area located in the north of Brazil (reference map in electronic supplementary material, figure S1). The numbers of confirmed malaria cases registered in this area from 2003 to 2020 were obtained from the SIVEP-Malaria database (Brazilian Ministry of Health) [1]. Cases included infections by *P. vivax* and *P. falciparum* which are endemic in the Brazilian Amazon area. The number of *P. vivax* or *P. falciparum* infections was aggregated by geographical unit, state or municipality, and unit of time, week or day. The population data from the Brazilian Institute of Geography and Statistics (IBGE) are used as a denominator to obtain incidence values.

We obtained climate data per municipality from the Database of Meteorological Data for Teaching and Research [30]. These data were also summarized in aggregated and mean values, whenever necessary and stated. Seasonality was evaluated for each state capital to cover all states and owing to data availability, when compared to the other municipalities of the same state.

### 2.2. Wavelet analysis

We used wavelet analysis to analyse incidence data, calculated as the ratio between the number of notified cases in the states and their populations, over time. After the application of the wavelet analysis, we analysed the wavelet power spectrum, altogether with the average power spectrum to better verify the seasonal relation of data.

The wavelet analysis uses mathematical functions that describe oscillation patterns in a given time interval to search for periodic conditions in the time series. For analysis of daily data, the lower period was defined as 1 day, and the upper period was defined as 730 days for weekly and monthly data, the limits of 1–104 weeks and 1–34 months were used, respectively.

To analyse the relationship between climate variables and notification data, a wavelet cross-power analysis relates data on malaria cases with temperature and rainfall data from each state capital.

The wavelet analysis uses the implementation from package WaveletComp [31], where the Morlet function [32] is the basis function translated and scaled, given by


(2.1) $$\psi \left(t\right)=\pi^{-1/4}e^{i\omega t}e^{-t^{2}/2},$$

where the rotation rate in radians per time unit ω (or angular frequency) is 6.

### 2.3. Modelling

The model used in this work to assess *P. vivax* notification data is derived from the model of Ayala & Villela [29], which considers the impact of pharmaceutical interventions in their modelling. We evaluate *P. vivax* notification data because cases of *P. vivax* malaria account for more than 75% of the total notified cases in the Brazilian Amazon basin region, as shown in electronic supplementary material, figure S3*a* by comparing the stratified data by parasites at any given time, and also reported by other authors [3]. The model from Ayala & Villela [29] analysed resistant and non-resistant (sensitive) strains and contains the state variables: $S_{h}$ for susceptible humans; $I_{vs}$ for individuals infected with sensitive strain; $I_{vr}$ for individuals infected with resistant strain; $P_{vs}$ for individuals infected with sensitive strain under drug treatment; $P_{vr}$ for individuals infected with resistant strain under drug treatment; $L_{vs}$ for individuals infected with sensitive strain and are with latent parasites; $L_{vr}$ for individuals infected with resistant strain and are with latent parasites. We provide the set of differential equations (equations (2.2)–(2.11)) in the adapted model where parameters are defined in table 1:

**Table 1.** Parameters used in the model in this work. Parameters with reference marked as ‘this work’ were estimated to better match the model to the malaria notified cases tested in this work.

parameter	description	value
*m*	ratio of mosquitoes per human $N_{m}/N_{h}$ (adimensional)	2435/625 [29]
*b*	probability of transmission of $I_{mv}$ to $S_{h}$ (adimensional)	0.0056 for Manaus, 0.0051 for Macapá (this work)
η	treatment coverage (adimensional)	0.05 for Manaus, 0.01 for Macapá (this work)
$\sigma_{v}$	proportion of infected symptomatic humans (adimensional)	0.33 (this work)
$r_{v}$	recovery rate of infected humans without treatment (day^−1^)	1/60 [29]
$\gamma_{v}$	progression rate from infected human to post-treatment stage (day^−1^)	1/9 [29]
ϕ	proportion of humans treated with primaquine (adimensional)	0.95 (this work)
$\phi_{t}$	probability of post-treatment human to progress to latent stage (adimensional)	0.21 [29]
$\phi_{u}$	probability of infected human to progress to latent stage (adimensional)	0.4 (this work)
κ	protective period of treatment (days)	30 [29]
$\epsilon_{v}$	infectious period of post-treatment human (days)	2.1 [29]
μ	birth and death rate of humans (day^−1^)	0.003 [29]
$c_{a}$	probability of transmission of asymptomatic to $S_{m}$ (adimensional)	0.12 (this work)
$c_{s}$	probability of transmission of symptomatic to $S_{m}$ (adimensional)	0.40 (this work)
Ψ	relapse rate of hypnozoites (day^−1^)	1/60 [29]
$\mu_{L}$	cleanse rate of hypnozoites (day^−1^)	1/425 [29]
($a_{1}$,$a_{2}$,$T_{0}$)	parameters for the biting rate equation	(0.000203, 11.7 ${\;}^{\circ }C^{-1}$, 42.3 ${\;}^{\circ }C$) [13]
($c_{1}$,$c_{2}$,$c_{3}$)	parameters for the Martens equation	(0.522 , 0.0367 ${\;}^{\circ }C^{-1}$, −0.000828 ${\;}^{\circ }C^{-2}$) [13]
($p_{1}$,$p_{2}$)	parameters for the daily survivorship of larvae	(0.0554 ${\;}^{\circ }C^{-1}$, −0.06737) [33]
$R_{max}$	maximum rainfall for survivorship of mosquitoes (mm)	33.0 for Manaus, 53.2 for Macapá (this work)
$p_{E}(R)$	daily survivorship for eggs (adimensional)	0.90 [15]
$p_{L}(R)$	daily survivorship for larvae (adimensional)	0.25 [15]
$p_{P}(R)$	daily survivorship for pupae (adimensional)	0.75 [15]
$b_{1}$, $b_{2}$, $b_{3}$	coefficients for the egg deposition rate equation	(−0.153 ${\;}^{\circ }C^{-2}$, 8.61 ${\;}^{\circ }C^{-1}$, −97.7) [15]


(2.2) $$\begin{array}{c} \frac{dS_{h}}{dt}=-ma\left(T\right)b\frac{I_{mvs}}{N_{m}}S_{h}-ma\left(T\right)b\left(1-\alpha \right)\frac{I_{mvr}}{N_{m}}S_{h}+\left(1-\eta \sigma_{v}\right)\left(1-\phi_{u}\right)r_{v}\left(I_{vs}+I_{vr}\right)\\ +\mu_{vl}\left(L_{vs}+L_{vr}\right)+\frac{\left[1-\phi_{t}\left(1-\phi \right)\right]}{\kappa }P_{vs}+\frac{\left[1-\phi_{t}\left(1-\phi \right)\right]}{\kappa \left(n+1\right)}P_{vr}, \end{array}$$

(2.3) $$\begin{array}{c} \frac{dI_{vs}}{dt}=ma\left(T\right)b\frac{I_{mvs}}{N_{m}}S_{h}-\left(1-\eta \sigma_{v}\right)r_{v}I_{vs}-\eta \sigma_{v}\gamma_{v}I_{vs}+\psi L_{vs}+ma\left(T\right)b\rho_{sr}\frac{I_{mvs}}{N_{m}}L_{vr}\\ +ma\left(T\right)b\frac{I_{mvs}}{N_{m}}L_{vs}+ma\left(T\right)b\left(1-\rho_{sr}\right)\frac{I_{mvr}}{N_{m}}L_{vs}, \end{array}$$

(2.4) $$\begin{array}{c} \frac{dL_{vs}}{dt}=\left(1-\eta \sigma_{v}\right)\phi_{u}r_{v}I_{vs}+\frac{\phi_{t}\left(1-\phi \right)}{\kappa }P_{vs}-\mu_{vl}L_{vs}-\psi L_{vs}-ma\left(T\right)b\frac{I_{mvs}}{N_{m}}L_{vs}\\ -ma\left(T\right)b\left(1-\alpha \right)\frac{I_{mvr}}{N_{m}}L_{vs}, \end{array}$$

(2.5) $$\frac{dP_{vs}}{dt}=\eta \sigma_{v}\gamma_{v}I_{vs}-\frac{P_{vs}}{\kappa },$$

(2.6) $$\begin{array}{c} \frac{dI_{vr}}{dt}=ma\left(T\right)b\left(1-\alpha \right)\frac{I_{mvs}}{N_{m}}S_{h}-\left(1-\eta \sigma_{v}\right)r_{v}I_{vr}-\frac{\eta \sigma_{v}\gamma_{v}}{n+1}I_{vr}+\psi L_{vr}+ma\left(T\right)b\left(1-\alpha \right)\frac{I_{mvr}}{N_{m}}L_{vr}\\ +ma\left(T\right)b\left(1-\alpha \right)\rho_{rs}\frac{I_{mvr}}{N_{m}}L_{vs}+ma\left(T\right)b\left(1-\rho_{sr}\right)\frac{I_{mvs}}{N_{m}}L_{vr}, \end{array}$$

(2.7) $$\begin{array}{c} \frac{dL_{vr}}{dt}=\left(1-\eta \sigma_{v}\right)\phi_{u}r_{v}I_{vr}+\frac{\phi_{t}\left(1-\phi \right)}{\kappa \left(n+1\right)}P_{vr}-\psi L_{vr}-\mu_{vl}L_{vr}-ma\left(T\right)b\left(1-\alpha \right)\frac{I_{mvr}}{N_{m}}L_{vr}\\ -ma\left(T\right)b\frac{I_{mvs}}{N_{m}}L_{vr}, \end{array}$$

(2.8) $$\frac{dP_{vr}}{dt}=\frac{\eta \sigma_{v}\gamma_{v}}{n+1}I_{vs}-\frac{P_{vr}}{\kappa \left(n+1\right)},$$

(2.9) $$\begin{array}{c} \frac{dS_{m}}{dt}=\Delta_{m}N_{m}-\left[a\left(T\right)c_{s}\sigma_{v}+a\left(T\right)c_{a}\left(1-\sigma_{v}\right)\right]\frac{I_{vs}}{N_{h}}S_{m}-a\left(T\right)c_{s}\frac{\epsilon_{v}}{\kappa }\left(1-\phi \right)\left(1-v\right)\frac{P_{vs}}{N_{h}}S_{m}\\ -\left[a\left(T\right)c_{s}\sigma_{v}+a\left(T\right)c_{a}\left(1-\sigma_{v}\right)\right]\left(1-\alpha \right)\frac{I_{vr}}{N_{h}}S_{m}-a\left(T\right)c_{s}\frac{\epsilon_{v}}{\kappa }\left(1-\alpha \right)\left(1-\phi \right)\frac{P_{vr}}{N_{h}}S_{m}\\ -a\left(T\right)c_{s}\frac{\epsilon }{\kappa }\left(1-\alpha \right)\left(1-\phi \right)v\frac{P_{vs}}{N_{h}}S_{m}-\mu_{m}\left(T\right)S_{m}, \end{array}$$

(2.10) $$\frac{dI_{mvs}}{dt}=\left[a\left(T\right)c_{s}\sigma_{v}+a\left(T\right)c_{a}\left(1-\sigma_{v}\right)\right]\frac{I_{vs}}{N_{h}}S_{m}+a\left(T\right)c_{s}\frac{\epsilon_{v}}{\kappa }\left(1-\phi \right)\left(1-v\right)\frac{P_{vs}}{N_{h}}S_{m}-\mu_{m}\left(T\right)I_{mvs},$$

(2.11) $$\begin{array}{c} \frac{dI_{mvr}}{dt}=\left[a\left(T\right)c_{s}\sigma_{v}+a\left(T\right)c_{a}\left(1-\sigma_{v}\right)\right]\left(1-\alpha \right)\frac{I_{vr}}{N_{h}}S_{m}+a\left(T\right)c_{s}\frac{\epsilon_{v}}{\kappa }\left(1-\alpha \right)\left(1-\phi \right)\frac{P_{vr}}{N_{h}}S_{m}\\ +a\left(T\right)c_{s}\frac{\epsilon }{\kappa }\left(1-\alpha \right)\left(1-\phi \right)v\frac{P_{vs}}{N_{h}}S_{m}-\mu_{m}\left(T\right)I_{mvr}, \end{array}$$

where


(2.12) $$N_{h}=S_{h}+I_{vs}+L_{vs}+P_{vs}+I_{vr}+L_{vr}+P_{vr},$$

and


(2.13) $$N_{m}=S_{m}+I_{mvs}+I_{mvr}.$$

The modification from the model by Ayala & Villela [29] relies on assuming that some parameters can be modelled as a function of temperature (T), namely the biting rate, the adult mosquito death ratio, the daily survivorship of larvae, the duration of the larvae stage and the egg daily deposition rate. In addition, other variables were considered as functions of rainfall (*R*), such as the daily survivorship of larvae, eggs and pupa.

The parameter describing the biting rate is a function of temperature as calculated [13] with the equation


(2.14) $$a\left(T\right)=a_{1}\left(T^{2}-a_{2}\;T\right)\sqrt{T_{0}-T},$$

where $a_{1}$, $a_{2}$ and $T_{0}$ are constants of the equation, and T is the temperature in °C. This equation represents a rising trend of daily biting rate with rising temperature up to a maximum of approximately 35°C, and is defined up to 42.3°C. The temperature series for municipalities in this study did not fall outside these limits.

The death rate of adult mosquitoes as a function of temperature was calculated with the Martens equation [33–35] re-calibrated by Mordecai *et al*. [13,33,34], which is given by


(2.15) $$\mu_{m}\left(T\right)=-\ln\left(c_{1}+c_{2}\;T+c_{3}\;T^{2}\right),$$

where the coefficients $c_{1}$, $c_{2}$ and $c_{3}$ are constants of the equation. This equation considers a constant relative humidity scenario, which is a reasonable approximation for the model since we are not considering these data in our model. The daily survivorship p(T) of adult mosquitoes was calculated with the relationship between the death rate and survivorship given by $p\left(T\right)=e^{-\mu_{m}\left(T\right)}$. Apart from the daily survivorship of adult mosquitoes, the daily survivorship of larvae was also a function of temperature [33,36], modelled by


(2.16) $$p_{L}\left(T\right)=e^{-(p_{1}T+p_{2})},$$

where $p_{1}$ and $p_{2}$ are constants of the equation , which were fitted by other authors to data [33,36]. The equation for $p_{L}(T)$ is derived from the relationship of the daily survivorship of larvae to the average larvae stage duration $t_{l}(T)=1/(p_{1}T+p_{2})$, given by $p_{L}\left(T\right)=e^{-1/t_{l}(T)}$.

The total survivorship of larvae stage is a product of its function of temperature $p_{L}(T)$ and its function of rainfall p(R), given by [15]


(2.17) $$p_{x}\left(R\right)=\left(\frac{4p_{M,x}}{R_{max}^{2}}\right)R\left(R_{max}-R\right),$$

where R is the rainfall in mm, x defines whether the function is calculated for eggs (E), larvae (L) or pupae (P), while $R_{max}$ and $p_{M,x}$ are constants of the equation. When the rainfall R value is above the $R_{max}$ value, the survivorship p(R) is set to 0. The value of $R_{max}$ represents the transition limit where rainfall stops being a positive factor in the increase of the population of vectors to becoming a negative factor owing to intense flooding which washes away the most immature life forms of the mosquito life cycle.

The egg deposition rate is also chosen to be a function of temperature [15], which is calculated through


(2.18) $$B_{E}\left(T\right)=\frac{b_{1}T^{2}+b_{2}T-b_{3}}{\mu_{m}\left(T\right)},$$

where $b_{1}$, $b_{2}$ and $b_{3}$ are constants of the equation. The total birth rate of the mosquitoes is given by [15]


(2.19) $$\Delta_{m}\left(T,R\right)=\frac{B_{E}\left(T\right)p_{L}\left(T\right)p_{L}\left(R\right)p_{E}\left(R\right)p_{P}\left(R\right)}{\tau_{L}}.$$

The necessary parameters to calculate the model with the added equations and their references are shown in table 1. We apply this model to the time series of cases observed in two of the municipalities studied with wavelet analysis, namely Macapá and Manaus, which had high association with climate variables and significant malaria incidence.

**Figure 1.**
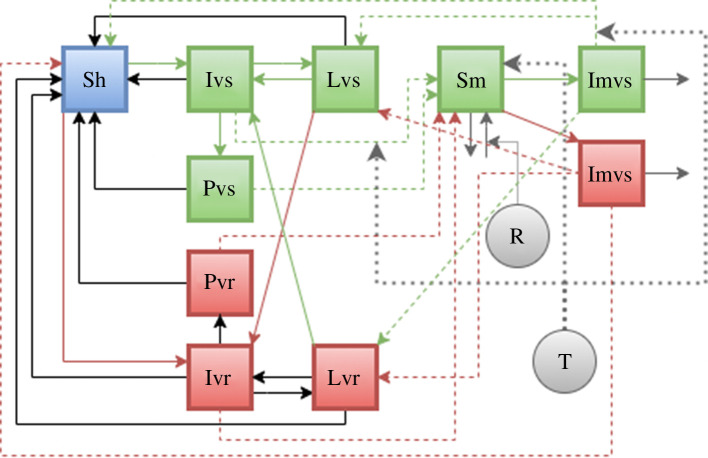
Compartmental model used in this work to model *P. vivax* malaria cases in the Brazilian Amazon region. All compartments, with the exception of ‘R’ and ‘T’ are the same as in [29]. The ‘R’ compartment shows which flows are influenced by rain, and the ‘T’ compartment shows which flows are affected by temperature.

The diagram for the compartmental model, together with the influence of rainfall and temperature, is shown in figure 1. The parameters that differ between different municipalities were chosen to be $R_{max}$, b and η, and also the initial population of selected municipalities of Macapá and Manaus. A few parameters (table 1) were used to fit this model to the time series of cases of *P. vivax* malaria in those municipalities.

### 2.4. Model ﬁtting

The model was fitted for each municipality separately. The parameters b, η and $R_{max}$ were fitted specifically for each municipality, while the parameters ϕ, $\sigma_{v}$, $\phi_{u}$, $c_{a}$ and $c_{s}$ were found to be independent of the municipality. To find the best value for each parameter, we calculated the time-series curve of new daily cases for the model, and minimized the mean absolute percentage error (MAPE) between the calculated curve with the model and the curve of notified cases using the Nelder–Mead simplex algorithm [37] with the following equation:


(2.20) $$\text{MAPE}=\sum_{i}^{N_{days}}\left|\frac{n_{i}^{model}-n_{i}^{notified}}{n_{i}^{notified}}\right|.$$

The parameters that minimized the above equation were then considered to calculate the final time-series curve of daily notified cases for the model presented in this work. Only the municipalities of Macapá and Manaus were selected to be presented in this work owing to their high number of notified cases and high association with the climactic variables as shown by the results of the wavelet analysis.

## 3. Results

### 3.1. Time series—malaria cases and climate variables

**Figure 2.**
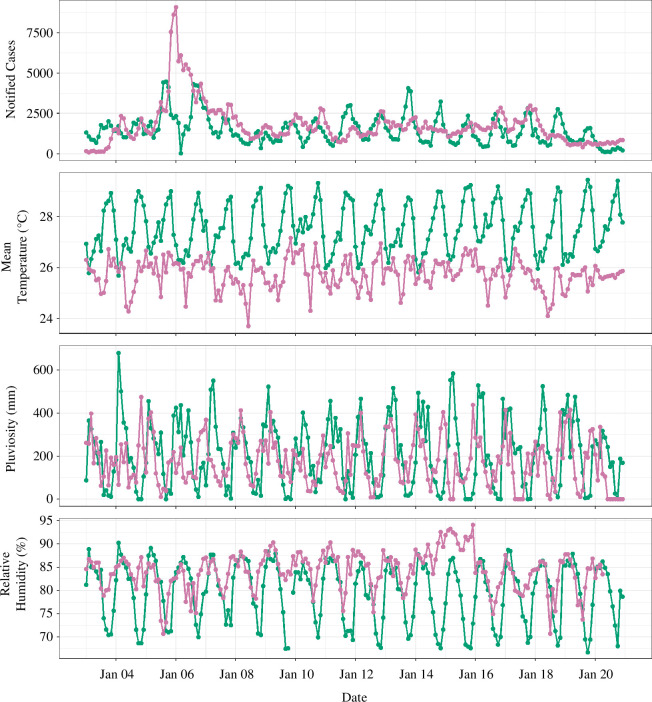
Macapá (green) and Cruzeiro do Sul (purple) notification data of *P. vivax* malaria cases and climate variables from January 2003 to December 2020. In the notified cases plot, Macapá data are multiplied by a factor of 5 for better comparison with the data from Cruzeiro do Sul.

The climate variables have cyclic patterns, which are shown in figure 2. Macapá and Cruzeiro do Sul have different patterns in both climate variables and notification data, with Macapá showing a more clear seasonal pattern. As presented in figure 2, Cruzeiro do Sul had a remarkable increase in notified cases in the year 2006, as also noted by other authors [38].

### 3.2. Wavelet power analysis

**Figure 3.**
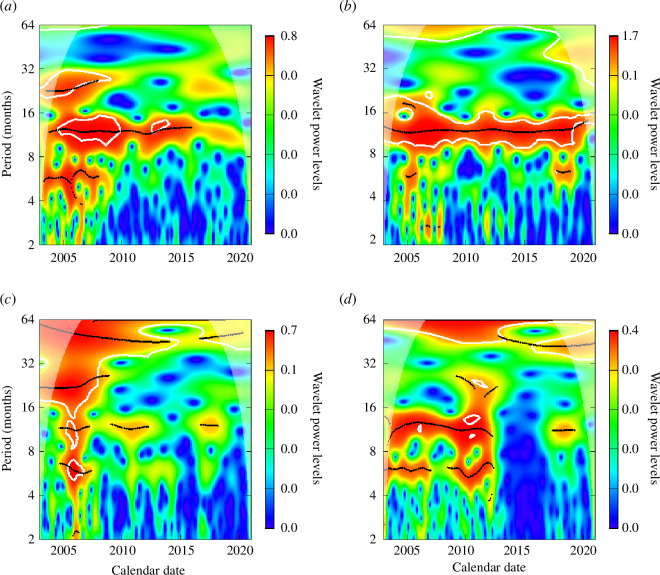
Monthly wavelet power spectra for malaria cases of infections by *P. vivax* in states of the Amazon region: Amazonas, Amapá, Acre and Pará (*a*–*d*). The red colour represents a higher wavelet power level, while the blue colour represents a lower wavelet power level, with the colours in between represented by the scale next to each panel.

The wavelet power spectra of notification data of four states are presented in figure 3. Amapá is the only state where the seasonality was very marked from 2005 to 2020, which is also clear when looking at the notification data. Other states have a very marked annual seasonality for a range of years, which is the case for Roraima, Pará, Rondônia and Maranhão which have shown a considerable annual seasonality up to the end of year 2014, and for Amazonas extending this period to the end of 2017. The states of Tocantins, Mato Grosso and Acre have sparse years with considerable seasonality, suggesting that the magnitude of seasonality within the notification data series has been lowered. The results of these states are presented in electronic supplementary material, figure S5.

### 3.3. Wavelet coherency analysis

Regarding the wavelet coherency analysis, we have analysed the following municipalities in the Amazon region: Manaus (state of Amazonas), Palmas (state of Tocantins), Cuiabá (state of Mato Grosso), Macapá (state of Amapá), Belém (state of Pará), Cruzeiro do Sul (state of Acre), Boa Vista (state of Roraima), Porto Velho (state of Rondônia) and São Luís (state of Maranhão). All these municipalities are the capitals of each state covering the geographical region of the Brazilian Amazon basin, except Cruzeiro do Sul. The main criterion for choosing these municipalities was the availability of data when compared to other municipalities in each state.

**Figure 4.**
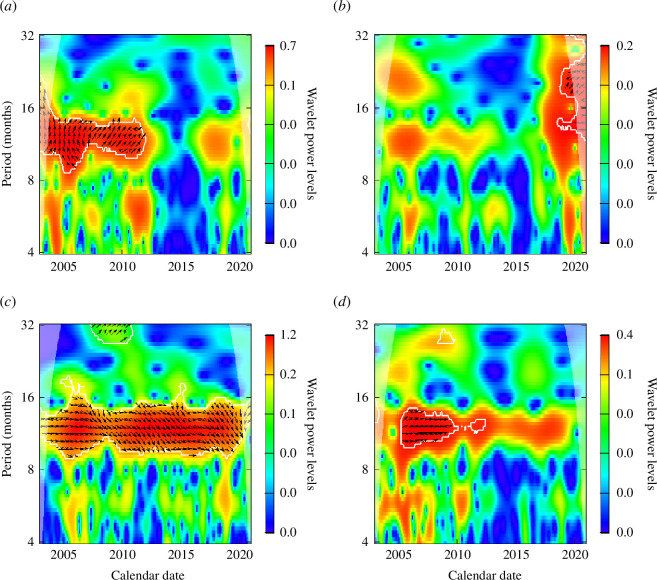
Wavelet coherency analysis with monthly notification data related to the monthly mean temperature for the capitals of each state of the Brazilian Amazon region: Belém, Cruzeiro do sul, Macapá and Manaus (*a*–*d*). The red colour represents a higher wavelet power level, while the blue colour represents a lower wavelet power level, with the colours in between represented by the scale next to each panel. The arrows indicate the direction of the effect, as the possible time lags between the different time series.

**Figure 5.**
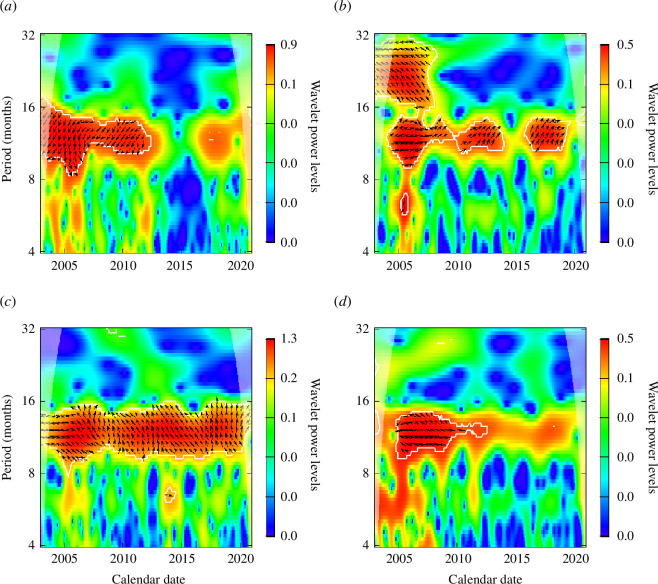
Wavelet coherency analysis with monthly notification data related to the monthly rainfall for the capitals of each state of the Brazilian Amazon region: Belém, Cruzeiro do sul, Macapá and Manaus (*a*–*d*). The red colour represents a higher wavelet power level, while the blue colour represents a lower wavelet power level, with the colours in between represented by the scale next to each panel. The arrows indicate the direction of the effect, as the possible time lags between the different time series.

The wavelet coherency analysis between notification data and temperature is shown in figure 4 and the analysis between notification data and rainfall is shown in figure 5. Coherency between cases and temperature was strong for a majority of municipalities in a yearly pattern and sparse semiannual periods. Despite some states showing decreasing seasonality of the notification data time series, the relation between the time series of temperature and notification data remains strong for a few municipalities. Results for the municipality of Belém exhibited this pattern with a strong cross-power effect between notification data and temperature for the year ranges of 2003–2014 and mid-2015–2020, while the seasonality of the notiﬁcation time series decreases around 2014, with no statistically significant seasonality thereafter. Results for other municipalities are presented in electronic supplementary material, figures S3 and S4.

Rainfall also had some sparse semiannual effects with notification data and significance within an annual cycle. As observed for the series of mean temperatures, the pattern along the years for the wavelet cross-power relation between rainfall and notification data does not necessarily follow the pattern of seasonality in varied periods and regions, such as observed for Boa Vista and Palmas (presented in electronic supplementary material, figure S4). The semiannual effects are seen mostly in the period before the year 2010, in which notified cases were more intense across the Brazilian Amazon region.

The phase difference of the wavelet cross-power analysis of mean temperature with notification data, and also between rainfall and notification data has followed almost the same pattern in all studied municipalities considering daily data (electronic supplementary material).

The varying seasonality found in the wavelet analysis justifies the inclusion of these climate data into our model owing to the high average coherence. It is also important to note the phase difference angle in the coherency analysis. As demonstrated in figures 4 and 5, both temperature and rainfall have their specific time-phase lags to better match their signals with notification data, a feature to be explored in the model. Also, each municipality has its time-phase lag difference, which implies that this is a factor to be municipality-specific, further reflecting the heterogeneity between the specific malaria dynamics in each municipality. Although malaria incidence seems associated with both rainfall and mean temperature, the phase patterns exhibited for each municipality varied, which needs to be considered during a modelling and surveillance stage.

### 3.4. Model fitting with times series from municipalities of the Amazon region

**Figure 6.**
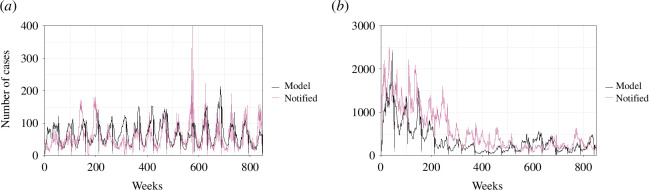
Modelling malaria notified cases by *P. vivax* using the model of this work: results for Macapá (*a*) and Manaus (*b*). A time lag period of 2 weeks was considered for temperature results, while for the rainfall, a time lag of 4 weeks was considered. The period for the analysis ranges from the beginning of 2003 to the end of October 2020.

The results concerning the application of the compartmental model are shown in figure 6. The model accurately captured the notification data for both the municipalities of Macapá and Manaus. Both municipalities were chosen to be analysed through this model because the coherence analysis provided stronger associations for these municipalities regarding the notified cases and the climate variables. These municipalities differ in the sense that Manaus has a mean downfall trend in the observed period, while Macapá presented a regular seasonal trend. Despite this difference, the model was capable of representing these trends by only using the different climate data from both municipalities.

## 4. Discussion

The presence of an annual cycle of malaria cases induces the presence of an epidemic peak every year, highlighting the need for continuous preparation of intervention measures, to hinder the spread of the disease. Malaria is still an endemic disease throughout many regions of the Brazilian Amazon region [39,40], and malaria cases are distributed very unequally [4]. Seasonality analyses reinforce these statements as they show that different states and municipalities have very different patterns throughout the years, which can be not only an impact of the environmental changes implied to these regions such as deforestation, change in land economical use and climate variations [27,41–43] but also owing to political and demographic aspects, such as internal and external migration [44–46].

The seasonality in the Brazilian Amazon region is very regional and heterogeneous across states. There is a clear yearly seasonality in the states of Amapá and Amazonas, whereas other states, such as Tocantins and Mato Grosso, have no clear seasonality. Many aspects contribute to this heterogeneity, ranging from environmental, to political aspects, such as the different hydrological regime of diverse areas [21,22] and policies [45]. Also, states like Mato Grosso and Tocantins had significantly lower incidences of malaria than the northern states. These observations reinforce the very important aspect of the need to research and understand malaria in Brazil at local levels, as the disease is still endemic and the spatial connection between areas where malaria is being successfully hindered and areas where malaria is still strong with annual epidemics is a challenge to public health surveillance. Therefore, considering heterogeneity is a need for public health malaria concerns towards elimination.

The seasonality of malaria incidence data is closely related to the seasonality of climate variables yearly. While not all regions exhibit a strong coherence between climate variables and notified cases for all the cases analysed, it is important to observe that different patterns and relationships are found throughout the Amazon region. These aspects are not unique to the Brazilian Amazon region, but other authors have explored it in other countries [24,33,47–49].

The climate variables are important drivers of malaria transmission. Not only the annual cycle but also the semiannual cycle of climate variables are important to understanding malaria transmission patterns. Also, it is suggested that climate and environmental changes are highly significant for malaria transmission, particularly owing to their close relationship.

Rainfall also has important yearly implications. The phase difference between the time series of rainfall and malaria-notified cases reveals a significant time lag, indicating an out-of-phase relationship, as demonstrated by wavelet coherency analysis. However, this anti-phasing relationship may require further investigation, as other studies have indicated an in-phase relationship for certain areas [17,21,36]. For mean temperature, in contrast to rainfall, effects are observed on both a semiannual and yearly basis, and these effects are in-phase with notification data.

Studies concerning the impact of climate variables on the population of the principal vector of malaria in the Amazon [50–53], *Anopheles darlingi*, have shown associations between rainfall and temperature with the vector population. These associations follow those on other regions of the world for other *Anopheles* sp. [54–57], and were also reflected by the present modelling, although equations were not necessarily derived for *A. darlingi*. The climate variables not only affect the density of vectors in a given area through daily survival rates [52] but also affect the activity of adult vectors transmitting the *Plasmodium*, e.g. via the biting rates.

Lana *et al*. [4] have demonstrated the heterogeneous nature of the Amazon region, revealing significantly different transmission settings across its municipalities. Our analysis underscores the importance of considering climate variables in understanding the complexity of malaria cases in the Amazon region, as the present results show considerable heterogeneity. Other conditions such as deforestation [43] and river levels across the Amazon region [22] may also have an impact and were not explored in this study. Despite the seasonal effects imposed by climatic variables, there is also the concern with sporadic environmental policies that may impact the number of notified cases without a proper study, such as the surge of cases in Cruzeiro do Sul in 2006 following a new policy encouraging the digging of fish tanks in this area, such as noted by Costa *et al*. [38], but without further investigation of this hypothesis.

The proposed model was capable of capturing the dynamics of the malaria incidence in both Manaus and Macapá regions. In this model, rainfall and temperature were important to describe the time series of malaria disease with the mechanistic model, as the wavelet analysis indicates the marked seasonality for these municipalities. Both temperature and rainfall have a marked influence on the vector cycle, and should therefore be considered in a model used in health surveillance planning. The time-lagged aspect between the climate variables and notification data is very important and may be an important driver to be studied in future studies regarding the prediction of outbreaks and surging notification.

The El Niño Southern Oscillation (ENSO) phenomenon is another considerable factor that has been shown to affect not only climate data, but also the malaria cycle [58–62]. Current evidence in South America suggests that El Niño has an effect in intensifying the annual cycle of malaria in regions such as Colombia [58,60,61] and Venezuela [63]. However, as suggested by Cabral *et al*. [64], the role of ENSO in malaria incidence might be more complex, as it may be a secondary effect that intensifies the climate variables, which then affect the malaria incidence through the fluctuations of the vector population. Flooding of rivers associated with ENSO might favour the population of vectors in dry regions, while droughts might favour the incidence of malaria in other regions such as Colombia [58,60,61]. Moreover, other works [61] did not present a relation between climate anomalies caused by ENSO and fluctuations in malaria incidence [61]. This study focused on the direct effects of temperature and rainfall on malaria incidence, not necessarily evaluating the ENSO influence on malaria incidence through climate anomalies, which might be investigated in future works.

The absence of climate data in certain areas requires data interpolation, which impacts the assessment of seasonality effects. Nevertheless, the methodology was applied to the municipalities, such as state capitals, with richer information in terms of climate data. The substantial heterogeneity in both malaria incidence and geographical patterns adds complexity to the problem [65–68]. The most recent Brazilian programme targeting malaria cases [6] aims to eliminate malaria cases and deaths by 2035, requiring continuous updates throughout its implementation for success. Despite numerous efforts in recent years, a resurgence in cases has been observed [4], emphasizing the need to not only monitor malaria-focused policies but also address broader issues such as deforestation, which has been on the rise in recent years [69].

Unlike previous models in the literature [15,33,48,70,71] that link climate variables, mosquito population dynamics and malaria cases, the model in this study incorporates a novel feature from Ayala & Villela [29] of introducing susceptible and resistant strains of the parasite. Therefore, this model also enables the modelling of changes in the drug application policy within the population. This capability enhances the model’s utility for assisting surveillance programmes.

Climate variables such as rainfall and temperature are intrinsically related to notified malaria cases in the Amazon region, where the seasonality of the environmental conditions influences the disease dynamics. Seasonality, however, might vary over large geographic areas. Estimations with mathematical models should particularly be applied in spatial units such as municipalities, particularly in areas with large incidences, where marked seasonality effects are more likely.

## Data Availability

Data and code can be accessed from the Zenodo repository [72]. Supplementary material is available online [73].
